# Insights into the Role of *Helicobacter pylori* Infection in Preeclampsia: From the Bench to the Bedside

**DOI:** 10.3389/fimmu.2014.00484

**Published:** 2014-10-09

**Authors:** Chiara Tersigni, Francesco Franceschi, Tullia Todros, Simona Cardaropoli, Giovanni Scambia, Nicoletta Di Simone

**Affiliations:** ^1^Department of Obstetrics and Gynecology, Università Cattolica Del Sacro Cuore, Policlinico A. Gemelli, Rome, Italy; ^2^Emergency Department, Università Cattolica Del Sacro Cuore, Policlinico A. Gemelli, Rome, Italy; ^3^Department of Surgical Sciences, Università degli Studi di Torino, Ospedale S. Anna, Turin, Italy

**Keywords:** preeclampsia, *Helicobacter pylori*, infection, placenta, anti-CagA antibody

## Abstract

Preeclampsia (PE) is defined as a hypertensive and coagulative disorder affecting about 2–8% of all pregnancies and is one of the main causes of maternal and fetal morbidity and mortality. Despite the great amount of studies run in this field, little is known about the precise pathogenic mechanisms behind PE. While endothelial and trophoblast dysfunctions, exaggerated inflammatory response, and hypercoagulative state have been shown to play a key role in the occurrence of PE, the primary trigger is still unknown. One of the hypotheses is that some infectious agents may represent a trigger for PE onset. Consistently, higher seroprevalence of *Helicobacter pylori* (HP) infection, a Gram-negative bacterium with a specific tropism for human gastric mucosa, has been shown in women with PE. Even tighter association has been found between PE and infection with cytotoxin-associated gene-A (CagA)-positive strains of HP. Recent *in vitro* studies have shown that anti-CagA antibodies cross-react with human trophoblast cells and determine a functional impairment in terms of cell invasiveness, thus, providing the first pathogenic model of HP infection-mediated placental damage. Since in the early process of implantation and placental development, trophoblast invasion of maternal decidua is a crucial step, the proposed autoimmune mechanism induced by HP infection, negatively interfering with the fetal side of the early developing placenta, may represent a mechanism explaining the higher seropositivity for HP infection among PE women. However, the contribution of HP infection to the pathogenesis of PE or to the worsening of its clinical presentation need to be further investigated as well as the possible impact of pre-pregnancy screening and eradication of HP infection on the incidence of the syndrome.

## Introduction

Preeclampsia (PE) is generally defined as new hypertension and substantial proteinuria at or after 20 weeks’ gestation (1). Complicating 2–8% of pregnancies, PE is a major cause of severe maternal morbidity and mortality and adverse perinatal outcomes worldwide (2, 3).

In the last 20 years, the incidence of PE has risen in the Western Countries, probably due to an increased prevalence of predisposing factors, such as advanced maternal age, chronic hypertension, diabetes, obesity, and the growing use of assisted reproductive techniques (4, 5).

Despite the great socio-economic impact of PE and the amount of studies carried out in this field, the pathogenic mechanisms leading to PE onset still remains unclear as well as an effective preventive intervention is still lacking (6).

The placental origins of PE have long been recognized and then formalized in the two stage model of the syndrome (7). The first stage is represented by inadequate development of the early placenta and its maternal blood supply, called poor placentation, which is established before 20 weeks and before clinical signs appear. During physiological placental development, extensive remodeling of maternal spiral arteries takes place in order to supply increased need of maternal blood in the second two trimesters of pregnancy. That process depends on extravillous cytotrophoblasts that invade the lining of the pregnant uterus from weeks 6 to 18 of gestation, expanding the vascular capacity of the utero-placental circulation (8). In many cases of PE, trophoblast invasion has been shown to be inadequate with poorly remodeled arteries and reduced capacity of the utero-placental circulation (9).

In the second stage, a dysfunctional and hypoxic placenta is considered to release factors into the maternal circulation that cause the clinical features of this condition, including hypertension and proteinuria, as well as clotting and liver dysfunction. These appear to arise from a generalized systemic inflammatory response, of which endothelial dysfunction is a prominent component (7).

Thus, nowadays, one of the biggest challenges in the research field of PE is to identify possible primary triggers of poor placentation, then leading to clinical PE, in order to develop effective preventative interventions. In that scenario, a possible role for infections has been widely suggested.

## Epidemiologic Association between *Helicobacter pylori* Infection and Preeclampsia

In the last few years, an epidemiological link between *Helicobacter pylori* (HP) infection and PE has been observed (10–13).

*Helicobacter pylori* is a Gram-negative bacterium with a specific tropism for the gastric mucosa (14); it is the main cause of chronic gastritis and peptic ulcer, as well as a risk factor for MALT-lymphoma and gastric cancer (15). Only some strains of HP possess determinants of pathogenicity, able to modulate the local and systemic inflammatory response (16), like the cytotoxin-associated gene-A (CagA), which encodes for a hydrophilic, surface-exposed protein (17). CagA-positive strains of HP have been shown to induce an inflammatory response in the gastric mucosa greater than that induced by CagA-negative ones (18). Owing to its capability to stimulate the immune system, HP has also been proposed to play a role in some extra-gastric diseases; in particular, the epidemiological association between HP infection and vascular diseases has been shown, including ischemic heart diseases, primary Raynaud’s phenomenon and migraine, all conditions characterized by endothelial dysfunction (19, 20).

Interestingly, anti-CagA antibodies seem to be able to cross-react with antigens localized on the surface of human endothelial cells in either normal or atherosclerotic arteries, thus providing a possible mechanism explaining this association (21, 22).

Daví and co-authors have shown an association between HP infection and high levels of *in vivo* markers of lipid peroxidation and platelet activation, urinary 8-iso-PGF2 and 11-dehydro-TXB2, respectively. Interestingly, successful eradication of HP infection led to a significant reduction in both markers, suggesting a novel mechanism by which an infectious agent could contribute to atherothrombosis (23).

A few years ago, Ponzetto et al. showed, for the first time, higher seropositivity for HP infection in 47 mothers with PE (51.1%) compared with 47 women with uneventful pregnancy (31.9%). The difference was even greater when considering positivity for CagA-positive strains of HP (80.9 and 14.9%, respectively) (10).

This epidemiologic association has subsequently been confirmed by several studies (11, 13) (Table 1), and a correlation between persistent and virulent infections (VacA/CagA seropositive patients) for HP and PE complicated by fetal intrauterine growth restriction (IUGR) has also been shown (13).

**Table 1:** **Studies investigating the prevalence of HP infection in general, and CagA+ strains HP infection, in particular, in healthy pregnant women (CTR) in comparison with preeclamptic women (PE)**.

Authors	Population (*n*)	HP+ (%)	*P*	CagA+ (%)	*P*
Ponzetto et al. (10)	CTR = 47	31.9		14.9	
	PE = 47	51.1	0.033	80.9	<0.001
UstUn et al. (11)	CTR = 40	12.5		–	–
	PE = 40	35.0	0.034	–	–
Pugliese et al. (12)	CTR = 25	32.0		28.0	
	PE = 25	84.0	<0.001	80.0	<0.001
Cardaropoli et al. (13)	CTR = 49	42.9		22.4	
	PE = 49	85.7	<0.001	81.6	<0.001

**P* < 0.05: statistically significant*.

Thus, since the association between HP infection and PE occurrence has been widely confirmed, we hypothesized that this bacterial infection might have a role as possible trigger in the etiopathogenesis of PE.

## Anti-CagA Antibodies Class IgG-Mediated Trophoblast Invasion Inhibition: An *In vitro* Model of HP-Induced Poor Placentation

To try to answer that question, we investigated whether HP infection might induce an immune humoral response able to trigger an autoantibody-mediated placental cellular damage. In particular, since anti-CagA antibodies are able to cross-react with antigens of endothelial cells (21) and cytotrophoblast cells show an endothelial origin, we tested murine anti-CagA antibodies class IgG – the only class of immunoglobulins able to cross placental barrier on human primary trophoblast cultures in order to find a possible cross-reaction. Interestingly, we observed that anti-CagA antibodies are able to bind, on the surface of trophoblast cells, to β-actin protein, one of the main components of cell cytoskeleton (24). Consistently, immunofluorescence performed on trophoblast cells using either anti-CagA or anti-β-actin antibodies showed an identical pattern of reaction, thus confirming β-actin to be the real cross-reacting protein. Interestingly, actin, in either endothelial or trophoblast cells, is not only important for maintaining the cell structure but it is also crucial for intercellular adhesion (25, 26) and it is now well established that actin-associated adhesions contribute to placental anchorage (26). We observed that anti-CagA antibodies show a dose-dependent binding activity as well and, as biological effect, a dose-dependent impairment of cytotrophoblast invasiveness *in vitro*, a crucial point for PE development. Furthermore, to better understand the molecular mechanisms involved in the antibody-mediated functional impairment of trophoblast cells, we examined the effect of anti-CagA on ERK activation and NF-kB nuclear translocation, two important factors activated during trophoblast proliferation, and we observed that anti-CagA antibodies are able to inhibit the activation of both elements (24).

**Figure 1:**
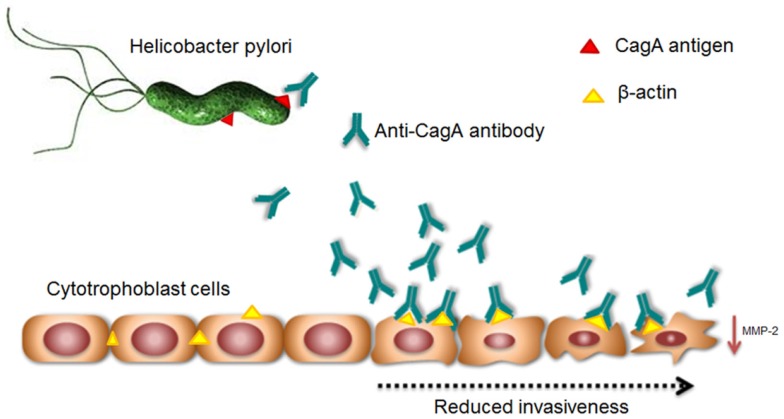
**Supposed mechanism of *Helicobacter pylori* infection-induced molecular mimicry leading to cross-reaction of anti-CagA antibodies to trophoblast cells and poor placentation**.

As a whole, these observations provided a possible autoimmune pathogenic mechanism induced by HP infection, negatively interfering with the fetal side of placental development. This pathogenic model of autoimmune-mediated placental impairment is the first one linking HP infection, poor placentation, and PE (Figure 1).

## Discussion

Although the cause of PE remains largely unknown, the leading hypotheses strongly rely on disturbed placental function early in pregnancy (27). Impaired remodeling of the spiral arteries has especially been considered as an early defect causing PE (28).

Inadequate placentation may lead to impaired intervillous perfusion and to the establishment of placental hypoxic status, causing oxidative stress of trophoblast cells and the release in maternal circulation of anti-angiogenic factors and trophoblast debris, believed to mediate maternal systemic inflammatory response, endothelial dysfunction, and hypercoagulability in PE syndrome (28, 29).

Several studies suggested a strong association between PE and HP infections (10–13). Our *in vitro* studies showed an anti-CagA antibody-mediated mechanism of placental impairment at fetal side of early placental development. Thus, it is likely that antibodies directed against bacterial CagA protein might cross-react with antigens expressed on trophoblast cell, and in particular with β-actin, through an immunologic mechanism called molecular mimicry, leading to autoimmune response. This binding could inhibit significantly trophoblast invasiveness, probably negatively interfering with intracellular signaling ad intercellular connections, potentially leading to inadequate placental development. That could represent an intriguing model of infection-induced autoimmune triggering for poor placentation and PE onset, giving an explanation to the higher prevalence of HP infection among women developing PE.

## Conclusion

More studies are needed to further investigate the impact of HP infection in triggering PE onset or, eventually, in worsening its clinical presentation.

However, it should be considered that, nowadays, in obstetrical practice, diseases with lower incidence than PE, like Rh alloimmunization or Down’s syndrome, are commonly screened. Thus, if HP will be confirmed as a contributing factor to PE, it will have important positive implications for the public health system, since the infection is treatable, and the future challenge would be to assess whether pre-pregnancy screening and preventive HP eradication would reduce the incidence of PE or moderate the severity of its clinical presentation.

## Author Contributions

Giovanni Scambia, Tullia Todros, Francesco Franceschi, Simona Cardaropoli, and Nicoletta Di Simone were responsible for the manuscript concept, design, and supervision. Chiara Tersigni performed the literature searches and extraction of data. Chiara Tersigni, Tullia Todros, and Nicoletta Di Simone drafted the manuscript.

## Conflict of Interest Statement

The authors declare that the research was conducted in the absence of any commercial or financial relationships that could be construed as a potential conflict of interest.

## References

[1] ACOG Committee on Practice Bulletins – Obstetrics. ACOG practice bulletin. Diagnosis and management of preeclampsia and eclampsia. Obstet Gynecol (2002) 99:159–67 10.1016/S0029-7844(01)01747-1 16175681

[2] Khan KS Wojdyla D Say L Gulmezoglu AM Van Look PFA WHO analysis of causes of maternal death: a systematic review. Lancet (2006) 367:1066–74 10.1016/S0140-6736(06)68397-9 16581405

[3] Duley L The global impact of pre-eclampsia and eclampsia. Semin Perinatol (2009) 33:130–7 10.1053/j.semperi.2009.02.010 19464502

[4] Berg CJ Mackay AP Qin C Callaghan WM Overview of maternal morbidity during hospitalization for labor and delivery in the United States: 1993–1997 and 2001–2005. Obstet Gynecol (2009) 113:1075–81 10.1097/AOG.0b013e3181a09fc0 19384123

[5] Wallis AB Saftlas AF Hsia J Atrash HK Secular trends in the rates of preeclampsia, eclampsia, and gestational hypertension, United States, 1987–2004. Am J Hypertens (2008) 21:521–6 10.1038/ajh.2008.20 18437143

[6] Chaiworapongsa T Chaemsaithong P Korzeniewski SJ Yeo L Romero R Pre-eclampsia part 2: prediction, prevention and management. Nat Rev Nephrol (2014) 10:531–40 10.1038/nrneph.2014.103 25003612 PMC5898797

[7] Redman CW Sargent IL Latest advances in understanding preeclampsia. Science (2005) 308:1592–4 10.1126/science.1111726 15947178

[8] Red-Horse K Zhou Y Genbacev O Prakobphol A Foulk R McMaster M Trophoblast differentiation during embryo implantation and formation of the maternal-fetal interface. J Clin Invest (2004) 114:744–54 10.1172/JCI200422991 15372095 PMC516273

[9] Redman CW Current topic: pre-eclampsia and the placenta. Placenta (1991) 12:301–8 10.1016/0143-4004(91)90339-H 1946241

[10] Ponzetto A Cardaropoli S Piccoli E Rolfo A Gennero L Kanduc D Pre-eclampsia is associated with *Helicobacter pylori* seropositivity in Italy. J Hypertens (2006) 24:2445–9 10.1097/HJH.0b013e3280109e8c 17082728

[11] UstUn Y Engin-UstUn Y Ozkaplan E Otlu B SaitTekerekoGlu M Association of *Helicobacter pylori* infection with systemic inflammation in preeclampsia. J Matern Fetal Neonatal Med (2010) 23:311–4 10.3109/14767050903121456 20222830

[12] Pugliese A Beltramo T Todros T Cardaropoli S Ponzetto A Interleukin-18 andgestosis: correlation with *Helicobacter pylori* seropositivity. Cell Biochem Funct (2008) 26:817–9 10.1002/cbf.1503 18777510

[13] Cardaropoli S Rolfo A Piazzese A Ponzetto A Todros T *Helicobacter pylori’s* virulence and infection persistence define pre-eclampsia complicated by fetal growth retardation. World J Gastroenterol (2011) 17:5156–65 10.3748/wjg.v17.i47.5156 22215939 PMC3243881

[14] Marshall BJ Barrett LJ Prakash C McCallum RW Guerrant RL Urea protects *Helicobacter* (Campylobacter) *pylori* from the bactericidal effect of acid. Gastroenterology (1990) 99:697–702 2379775 10.1016/0016-5085(90)90957-3

[15] Gasbarrini G Malfertheiner P Deltenre M Mégraud F O’Morain C Pajares-García J New concepts concerning management of *Helicobacter pylori* infection: 2 years after the Maastricht consensus report. Ital J Gastroenterol Hepatol (1998) 30:S244–7 10077746

[16] Crabtree JE Kersulyte D Li SD Lindley IJ Berg DE Modulation of *Helicobacter pylori* induced interleukin-8 synthesis in gastric epithelial cells mediated by cag PAI encoded VirD4 homologue. J Clin Pathol (1999) 52:653–7 10.1136/jcp.52.9.653 10655985 PMC501539

[17] Nguyen LT Uchida T Tsukamoto Y Trinh TD Ta L Mai HB Clinical relevance of cag PAI intactness in *Helicobacter pylori* isolates from Vietnam. Eur J Clin Microbiol Infect Dis (2010) 29:651–60 10.1007/s10096-010-0909-z 20372956 PMC3137892

[18] Sugimoto M Ohno T Graham DY Yamaoka Y Gastric mucosal interleukin-17 and-18 mRNA expression in *Helicobacter pylori* induced *Mongolian gerbils*. Cancer Sci (2009) 100:2152–9 10.1111/j.1349-7006.2009.01291.x 19694753 PMC3128813

[19] Pellicano R Franceschi F Saracco G Fagoonee S Roccarina D Gasbarrini A *Helicobacters* and extragastric diseases. Helicobacter (2009) 14:58–68 10.1111/j.1523-5378.2009.00699.x 19712170

[20] Franceschi F Gasbarrini A *Helicobacter pylori* and extragastric diseases. Best Pract Res Clin Gastroenterol (2007) 2:325–34 10.1016/j.bpg.2006.10.003 17382280

[21] Franceschi F Sepulveda AR Gasbarrini A Pola P Silveri NG Gasbarrini G Cross-reactivity of anti-CagA antibodies with vascular wall antigens: possible pathogenic link between *Helicobacter pylori* infection and atherosclerosis. Circulation (2002) 106:430–4 10.1161/01.CIR.0000024100.90140.19 12135941

[22] Franceschi F Niccoli G Ferrante G Gasbarrini A Baldi A Candelli M CagA antigen of *Helicobacter pylori* and coronary instability: insight from a clinico-pathological study and a meta-analysis of 4241 cases. Atherosclerosis (2009) 202:535–42 10.1016/j.atherosclerosis.2008.04.051 18599062

[23] Daví G Neri M Falco A Festi D Taraborelli T Ciabattoni G *Helicobacter pylori* infection causes persistent platelet activation in vivo through enhanced lipid peroxidation. Arterioscler Thromb Vasc Biol (2005) 25:246–51 10.1161/01.ATV.0000147128.10278.99 15472127

[24] Franceschi F Di Simone N D’Ippolito S Castellani R Di Nicuolo F Gasbarrini G Antibodies anti-CagA cross-react with trophoblast cells: a risk factor for pre-eclampsia? Helicobacter (2012) 17:426–34 10.1111/j.1523-5378.2012.00966.x 23066738 PMC3739447

[25] Pardridge WM Nowlin DM Choi TB Yang J Calaycay J Shively JE Brain capillary 46,000 dalton protein is cytoplasmic actin and is localized to endothelial plasma membrane. J Cereb Blood Flow Metab (1989) 9:675–80 10.1038/jcbfm.1989.95 2777936

[26] Aplin JD Jones CJP Harris LK Adhesion molecules in human trophoblast – a review. I. Villous trophoblast. Placenta (2009) 30:293–8 10.1016/j.placenta.2008.12.001 19131106

[27] Steegers EAP von Dadelszen P Duvekot JJ Pijnenborg R Pre-eclampsia. Lancet (2010) 376:631–44 10.1016/S0140-6736(10)60279-6 20598363

[28] Brosens I Robertson WB Dixon HG The role of spiral arteries in the pathogenesis of preeclampsia. In: Wynn RM, editor. Obstetrics and Gynecology Annual. New York: Appleton-Century-Crofts (1972). p. 177–91 4669123

[29] Redman CW Sargent IL Staff ACIFPA Senior award lecture: making sense of pre-eclampsia - two placental causes of preeclampsia? Placenta (2014) 35:S20–5 10.1016/j.placenta.2013.12.008 24477207

